# R‐Loops Mediated Odontogenic Differentiation of hDPSCs by Activating the cGAS‐STING Pathway Under Inflammatory Microenvironment

**DOI:** 10.1111/odi.70379

**Published:** 2026-06-12

**Authors:** Yun Yang, Yiqing Wang, Yating Miao, Zhipu Luo, Lin Niu, Ruirui Liu

**Affiliations:** ^1^ Key Laboratory of Shaanxi Province for Craniofacial Precision Medicine Research, College of Stomatology Xi'an Jiaotong University Xi'an Shaanxi Province China; ^2^ Wuhan Wuchang Hospital Wuhan Hubei Province China; ^3^ Clinical Research Center of Shaanxi Province for Dental and Maxillofacial Diseases, Department of Prosthodontics, College of Stomatology Xi'an Jiaotong University Xi'an Shaanxi Province China

**Keywords:** cGAS‐STING, dental pulp stem cell, odontogenic differentiation, pulpo‐dentinal complex, R‐loops

## Abstract

**Background:**

R‐Loops are three‐stranded DNA/RNA hybrids implicated in immune responses. Their role in mesenchymal stem cell differentiation, particularly in dental pulp regeneration under inflammation, remains unclear.

**Methods:**

Rat pulpo‐dentinal complex (PDC) injury models and LPS‐stimulated human dental pulp stem cells (hDPSCs) were used. R‐Loops and cGAS‐STING were evaluated via immunofluorescence, immunohistochemistry, RNA‐seq, RT‐qPCR, ALP, and Alizarin Red S staining. Interventions included RNase H1 overexpression (to degrade R‐Loops) and STING inhibitor H151.

**Results:**

Inflammation induced R‐Loops accumulation and cGAS‐STING activation in vivo and in vitro, correlating with impaired odontogenic differentiation. RNase H1 or H151 treatment downregulated inflammatory cytokines (IFN β, IL 6, TNF α) and restored mineralization capacity of hDPSCs.

**Conclusion:**

R‐Loops promote inflammation and suppress odontogenesis via cGAS‐STING signaling. Targeting this axis alleviates inflammation and enhances regenerative potential, offering a therapeutic strategy for pulpitis.

## 1 Introduction

The pulpo‐dentinal complex (PDC), a unique configuration encompassing the intricate relationship between dental pulp and surrounding dentin tissue, works as a sophisticated biological unit in immune defense and tissue repair following mechanical trauma or microbial insults (Murray and Garcia‐Godoy 2004). Regenerative endodontics aims to restore PDC integrity, yet persistent inflammation often induces hDPSCs (the resident mesenchymal progenitors) dysfunction and compromises their mediated repair (Spagnuolo et al. 2023).

cGAS‐STING axis, an evolutionarily conserved innate immune pathway, is implicated in surveilling the abnormal DNA from pathogens or damaged cells and operates as a molecular rheostat balancing tissue repair and immunopathology (Decout et al. 2021). Mechanistically, cGAS undergoes liquid–liquid phase separation upon binding cytosolic dsDNA fragments ≥ 45 bp, catalyzing 2′3′‐cGAMP synthesis that allosterically activates STING oligomerization on the endoplasmic reticulum (ER) membrane (Kwon and Bakhoum 2020). This interaction triggers TBK1‐mediated IRF3 phosphorylation (Ser386) and non‐canonical NF κB activation via TRAF3/6, driving transcription of IFN β and IL 6 through stochastic enhancer‐promoter looping (Mao et al. 2024). Dysregulation of the cGAS‐STING pathway is implicated in various disorders, including autoimmune diseases, cancer, and neurological conditions (Chen and Xu 2023). Thus, a deeper understanding of the cGAS‐STING axis offers valuable insights for developing novel therapeutic strategies for treating a range of conditions.

R‐Loops are recognized as spatial organizers of 3D genome architecture and typically found at unmethylated CpG islands, highly transcribed regions and repetitive sequences (Duardo et al. 2024; Ginno et al. 2012). Physiological R‐Loops are involved in immunoglobulin (Ig) class‐switch recombination, mammalian mitochondrial DNA replication, transcription termination and DNA methylation (García‐Muse and Aguilera 2019). While pathological R‐Loops accumulation can disrupt replication, cause DNA breaks, and threaten genome stability and link R‐Loops to inflammation and immune responses, with mutations in R‐Loop regulators implicated in autoimmune disorders (García‐Muse and Aguilera 2019; Lim et al. 2015; Günther et al. 2015; Zhao et al. 2023; Uruci et al. 2021). Research indicated that LPS treatment in HEK 293T cells induced R‐Loops increase at the ENPP2 gene's transcription start site, gene body, and a negative locus (Argaud et al. 2019). Excessive nuclear R‐Loops could cause RNA–DNA hybrids to enter the cytoplasm, activating the cGAS‐STING pathway and triggering innate immunity (Crossley et al. 2023).

Here we propose that the cGAS‐STING axis links LPS‐induced R‐Loops to metabolic inflexibility in hDPSCs. Disrupting this axis may reduce inflammation and partially restore hDPSCs' differentiation and odontogenic ability. It could be a therapeutic target for PDC regeneration through an “epigenetic‐metabolic checkpoint” paradigm, proposing chronotherapeutic strategies to uncouple protective inflammation from degenerative signaling.

## 2 Materials and Methods

### 2.1 Construction of Rat Pulpo‐Dentinal Complex‐Injury Models

All animal studies were conducted with the approval of the Medical Ethics Committee of Stomatology Hospital of Xi'an Jiaotong University. The project accords with the requirements of medical and teaching ethics and is approved to conduct. The ethics approval reference number for the study is [2022]005, Date of the ethics approval was May 10, 2022.

Twenty‐four male Sprague–Dawley rats (4‐week‐old) weighing from 180 to 220 g were selected, anesthetized with 2% isoflurane inhalation. After sterilizing the maxillary molars with 75% ethanol, a 1 mm‐depth round cavity was prepared with a #1/4 round bur (TC‐21EF, Japan) on occlusal surfaces of the left mandibular first molars as the dentin defect group (DD group), while the pulp of the right mandibular first molar was ground until pulp perforation as the pulp perforation group (PP group). The rats were sacrificed 0, 1, 7, and 14 days after surgery and anesthetized via isoflurane inhalation and cervical dislocation. The work has been reported in line with the ARRIVE guidelines 2.0.

### 2.2 Immunofluorescence (IF) Staining

The whole mandibles of rats were taken out and fixed in 4% paraformaldehyde (PFA) (Boster, China). After decalcified with 10% ethylenediaminetetraacetic acid (EDTA) (PH = 7.4) (Boster, China) for 1 month, the tissues were embedded in paraffin and trimmed into 5‐μm‐thick sections by microtome (Leica, Germany). Immunofluorescence staining was performed according to a previous procedure (Huang et al. 2022), anti‐DNA–RNA Hybrid (Kerafast; 1:250; cat# ENH001) primary antibodies were used and the pictures were detected under a Fluorview FV300 laser scanning confocal microscope (Olympus, Japan).

### 2.3 Immunohistochemistry (IHC) Staining

Immunohistochemical staining was performed using Rabbit/Mouse IgG immunohistochemistry kit (Boster, China) according to the manufacturer's instructions and previous procedure (Xiao et al. 2023). The following primary antibodies were used: anti‐γ H2AX (Abcam; 1:200; cat# ab81299‐10), anti‐cGAS (Proteintech; 1:200; cat# 26416‐AP), anti‐STING (Proteintech; 1:500; cat# 66680‐1‐Ig).

### 2.4 Human Dental Pulp Stem Cells (hDPSCs) Culture

Healthy human dental pulp tissue was gently isolated from freshly extracted impacted third mandibular molars or premolars for orthodontic therapy from healthy donors (≤ 25 years old). The patient and their guardian provided a written informed consent form for the dental sample. hDPSCs were isolated by tissue enzyme digestion method. hDPSCs at passages 2–5 were used for the following experiments.

### 2.5 RNA‐Sequencing (RNA‐Seq) Analysis

Following 20 μg/mL LPS (Sigma, America) stimulating for 1 day, total RNAs were extracted via Trizol reagent (Thermofisher, America) and mRNA captured by oligo(dT) magnetic beads (Thermofisher, America) was fractured by NEBNextR Magnesium RNA Fragmentation Module (Thermofisher, America). Next, generated cDNA libraries and sequenced using illumina NovaseqTM 6000 (LC Bio Technology CO. Ltd. Hangzhou, China). Genes changed more than twofold between two groups and *p* value < 0.05 were referred to as differentially expressed genes. The identified genes were used for gene ontology (GO) and Kyoto Encyclopedia of Genes and Genomes (KEGG) pathways analysis. The raw data of RNA‐sequencing were verified by MD5 and deposited in the NCBI Gene Expression Omnibus.

### 2.6 Lentivirus Transfection

RNase H1 overexpression lentiviral vectors (encodes enzymes capable of eliminating R‐Loops through selectively degrading RNA moiety within DNA–RNA hybrid (Suzuki et al. 2010; Paulsen et al. 2009)) and empty vectors (no RNase H1 insert) were purchased from GenePharma Co. Lentivirus was induced into culture medium following the protocol of the manufacturer. Empty virus vector GFP served as a negative control. Specifically, after hDPSCs were cultured to reach 30%–40% confluence, appropriate amounts of lentivirus according to the optimal MOI were transfected with polybrene. The expression of RNase H1 was then assessed by RT‐qPCR and Western blotting.

### 2.7 Real‐Time Polymerase Chain Reaction (RT‐qPCR)

Total RNA was extracted by Trizol (Thermofisher, America) and reversely transcribed into cDNA with 5× Evo M‐MLV RT Reaction Mix (Accurate Biology, America) following the manufacturer's protocol. Real‐time reverse transcription polymerase chain reaction was performed with SYBR Green PCR kit (Accurate Biology, America) according to the manufacturer's instructions (Table 1).

**TABLE 1:** Sequences of the primers for real‐time polymerase chain reaction.

Gene	Sequence
H‐GAPDH‐F	TGTGTCCGTCGTGGATCTGA
H‐GAPDH‐R	TTGCTGTTGAAGTCGCAGGAG
H‐IFN β‐F	ACCAACAAGTGTCTCCTCCAAAT
H‐IFN β‐R	GCAGTATTCAAGCCTCCCATTCA
H‐TNF α‐F	CTGCCTGCTGCACTTTGGAG
H‐TNF α‐R	ACATGGGCTACAGGCTTGTCACT
H‐IL 6‐F	TCCAGTTGCCTTCTTGGGAC
H‐IL 6‐R	GTACTCCAGAAGACCAGAGG
H‐RNase H1‐F	CAGAGCCGTATGCAAAGCAC
H‐RNase H1‐R	GACGACGAAGTCTCCCATGT
H‐cGAS‐F	GCCCTGCTGTAACACTTCTTATT
H‐cGAS‐R	GATAGCCGCCATGTTTCTTCTTG
H‐STING‐F	CTGTTGCTGCTGTCCATCTATTT
H‐STING‐R	AGCCGCAGATATCCGATGTAATA
H‐OCN‐F	GGCAGCGAGGTAGTGAAGAGA
H‐OCN‐R	CTCCTGAAAGCCGATGTGG
H‐DMP1‐F	TGGTCCCAGCAGTGAGTCCA
H‐DMP1‐R	TGTGTGCGAGCTGTCCTCCT
H‐RUNX2‐F	TGGTTACTGTCATGGCGGGTA
H‐RUNX2‐R	TCTCAGATCGTTGAACCTTGCTA
H‐BMP2‐F	ACCCGCTGTCTTCTAGCGT
H‐BMP2‐R	TTTCAGGCCGAACATGCTGAG

### 2.8 Western Blot Analysis

Proteins from hDPSCs were extracted using RIPA buffer (Beyotime, China) supplemented with PMSF (Beyotime, China) and protease inhibitor (Beyotime, China). Denatured proteins were subjected to SDS/PAGE in 8%–12% gradient gel (Boster, China) and then transferred to the PVDF membrane (Millipore, America). The membranes were blocked with 5% milk or BSA and then incubated with a primary antibody overnight at 4°C. After incubation with appropriate horseradish peroxidase‐conjugated goat anti‐rabbit IgG (Boster; 1:5000; cat# BA1054) or goat anti‐mouse IgG (Boster; 1:5000; cat# BA1050) for 60 min, the images were visualized by ECL Hypersensitivity Luminescence Kit (Boster, China) and exposed to a film. The data were normalized by developing the β‐actin as a loading control. The following primary antibodies were used: anti‐cGAS (Proteintech; 1:5000; cat# 26416‐AP), anti‐STING (Proteintech; 1:5000; cat# 66680–1‐Ig), anti‐pTBK1 (Cell Signaling Technology; 1:1000; cat# 5483 T), anti‐TBK1 (Cell Signaling Technology; 1:1000; cat# 3504T), anti‐β‐actin (Proteintech; 1:20000; cat# 66009‐1‐Ig).

### 2.9 Alkaline Phosphatase and Alizarin Red S Staining

After the hDPSCs reached nearly 70%–80% confluence, the medium was replaced by odontogenic medium (OM) containing 10% FBS (Gibco, America), 1% penicillin/streptomycin (Gibco, America), 10 mM sodium β‐glycerophosphate (Sigma, America), 0.1 μM dexamethasone (Sigma, America) and 50 μg/mL ascorbic acid (Sigma, America) and supplemented with 20 μg/mL LPS. The medium was changed every 2–3 days. After 7 and 21 days, hDPSCs were stained with the BCIP/NBT ALP Colorimetric Kit and ARS solution according to the manufacturer's instructions and the absorbance was detected at 520 and 562 nm respectively.

### 2.10 Statistical Analysis

Statistical analysis was performed using Student's *t*‐tests or one‐way analysis of variance at a significance level of *p* < 0.05 using GraphPad Prism 8. All data are presented as the means ± standard deviations.

## 3 Results

### 3.1 Inflammation Stimulation Induced DNA Breaks and Elevated R‐Loops

γ H2AX, a hallmark of DNA double strand breaks (DSBs), remarkably increased in the dentin defect group (DD group) at 1, 7, and 14 days (Figure 1a). In the pulp exposure group (PP group), the positive staining of γ H2AX in the pulp tissue increased significantly at a later stage (7 and 14 days) (Figure 1a).

**FIGURE 1:**
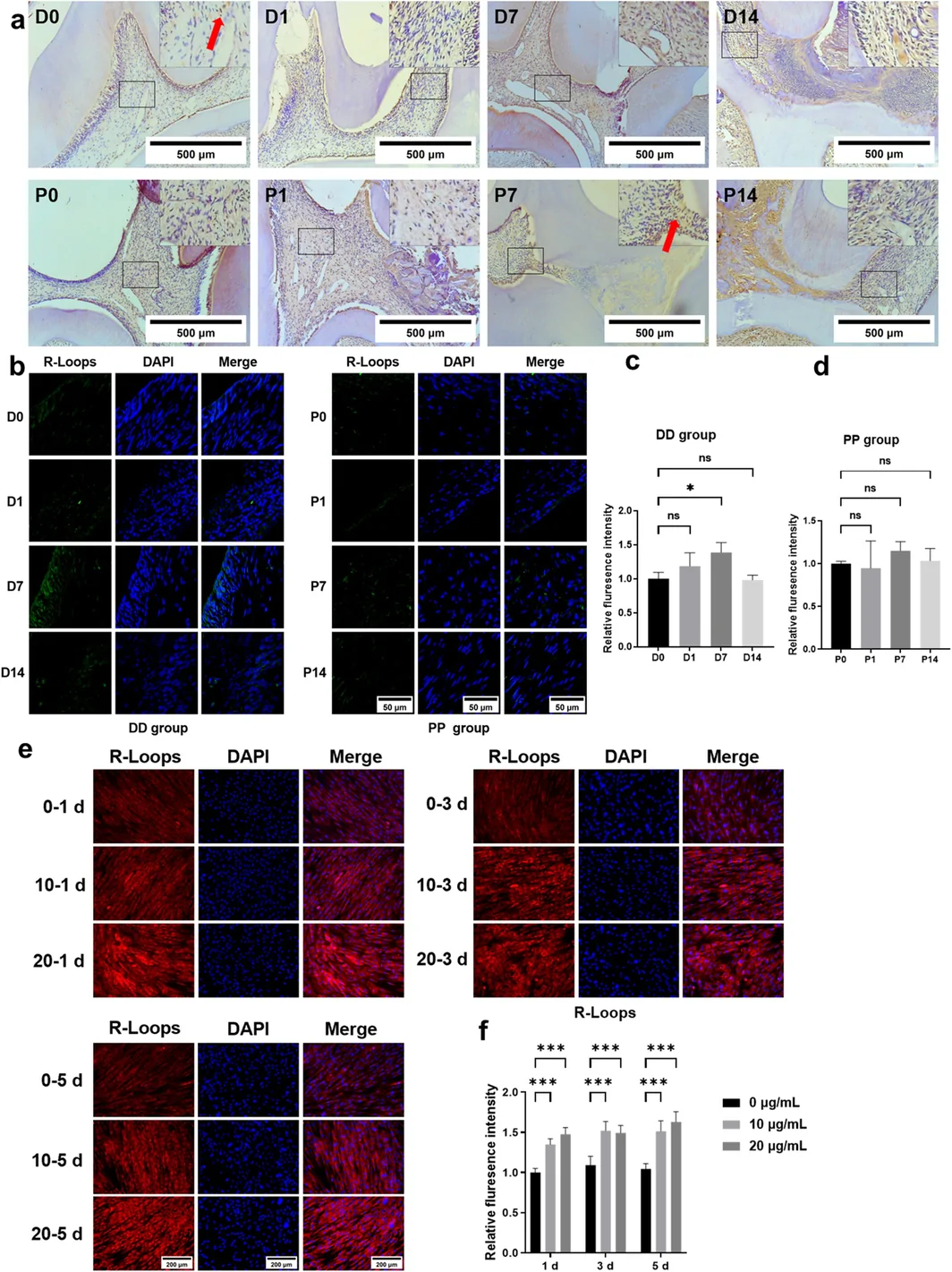
Inflammation stimulation induced DNA break and elevated R‐Loops (a) IHC staining of γ H2AX expression in rat dental pulp tissue on 0, 1, 7, and 14 days post‐operation. (b–d) IF staining of R‐Loops and quantitative analysis in rat dental pulp tissue on 0, 1, 7, and 14 days post‐operation. (e, f) IF staining of R‐Loops and quantitative analysis after hDPSCs stimulated with gradient concentration LPS (0, 10, 20 μg/mL) for 1, 3, and 5 days (significance was determined via one‐way ANOVA analysis of variance; data are represented as mean ± SD. ns: *p* > 0.05, *p < 0.05, **p < 0.01, ***p < 0.001).

Immunofluorescence staining using the S9.6 monoclonal antibody which targets DNA: RNA hybrids in R‐Loops was conducted for R‐Loops identification (Boguslawski et al. 1986). Results showed that R‐Loops levels were significantly upregulated on 7 days (*p <* 0.05) and then declined on 14 days (Figure 1b,c). In PP group, R‐Loops elevated on 7 days but without statistical differences (Figure 1b,d) (*p* > 0.05). Similarly, after hDPSCs were stimulated with gradient concentration LPS (0, 10, 20 μg/mL) for 1, 3, and 5 days in vitro, a higher fluorescence intensity of R‐Loops was observed (Figure 1e,f). The results indicated that PDC injury and inflammation stimulation induced DNA break and elevated R‐Loops accumulation.

### 3.2 Inflammatory Microenvironment Activated cGAS‐STING Pathway

RNA‐Seq indicated that innate immune response, immune system response, and defense response to virus were the three top main upregulated branches in the biological process branch (Figure S1a). Moreover, the cytosolic DNA‐sensing pathway was enriched besides classical inflammatory pathways such as the TNF signaling pathway, NF κB signaling pathway, IL 17 signaling pathway, and etc. (Figure 2a). cGAS‐STING pathway is an evolutionarily conserved antiviral immune and DNA‐sensing pathway, suggesting it was likely activated under PDC injury inflammatory microenvironment.

**FIGURE 2:**
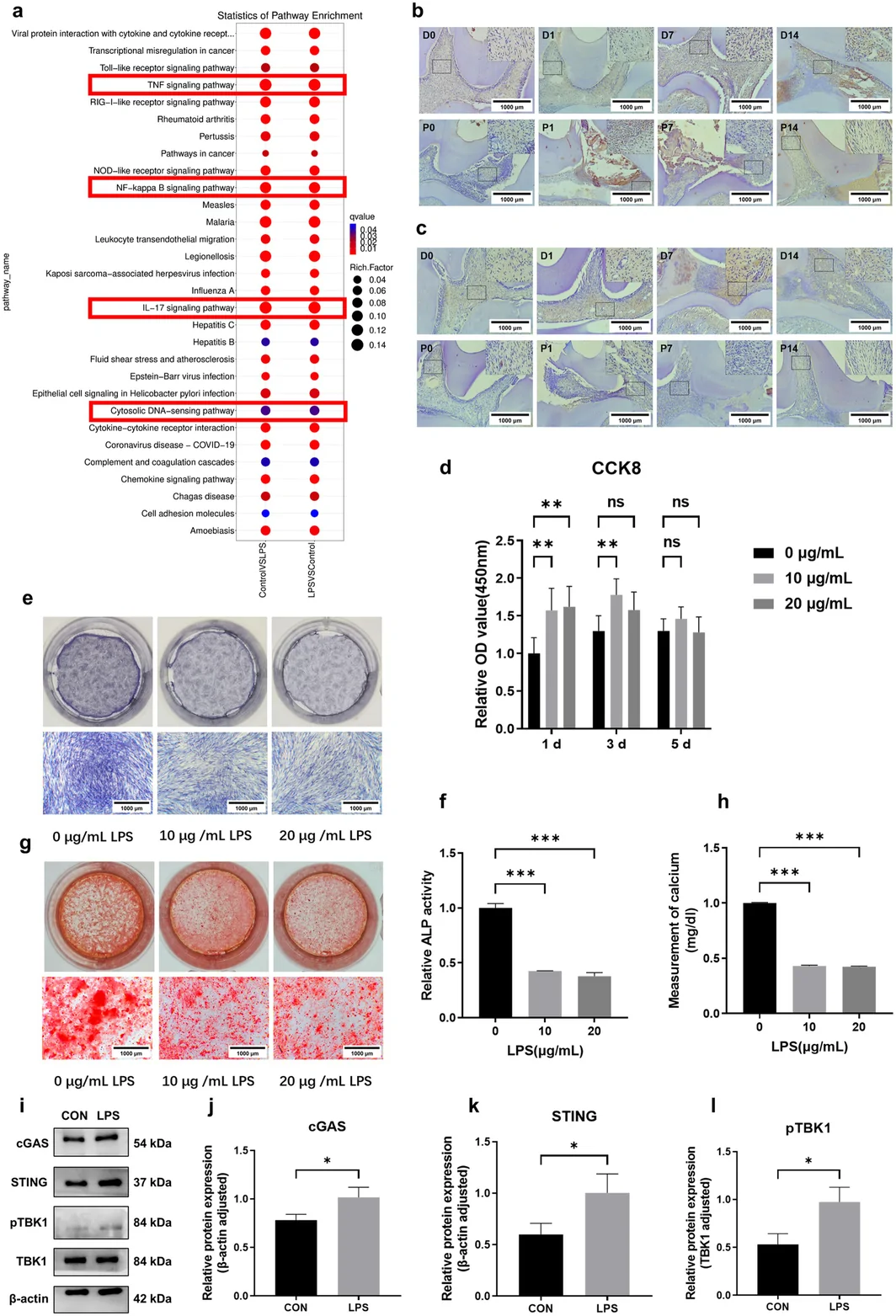
Inflammatory microenvironment activated cGAS‐STING pathway (a) KEGG pathway enrichment analysis of the differentially expressed genes between control and LPS group (20 μg/mL for 1 day). (b) IHC staining of cGAS expression in rat dental pulp tissue on 0, 1, 7, and 14 days post‐operation. (c) IHC staining of STING expression in rat dental pulp tissue on 0, 1, 7, and 14 days post‐operation. (d) The viability of hDPSCs stimulated by different gradient concentration LPS (0, 10, 20 μg/mL) for 1, 3, and 5 days. (e, f) ALP staining and quantitative analysis of hDPSCs stimulated by different gradient concentration LPS (0, 10, 20 μg/mL) for 7 days. (g, h) Alizarin red S staining and quantitative analysis of hDPSCs stimulated by different gradient concentration LPS (0, 10, 20 μg/mL) for 21 days. (i–l) The protein expression levels of cGAS‐STING pathway molecules (cGAS, STING, pTBK1, TBK1) examined by Western blot (significance was determined via one‐way ANOVA analysis and *t*′‐test analysis of variance; data are represented as mean ± SD. ns: *p* > 0.05, *p < 0.05, **p < 0.01, ***p < 0.001).

In Rat PDC inflammation model, we found cGAS was significantly increased with progressive exacerbation on D1, D7, D14, P1, P7, P14 compared with control D0 and P0 (Figure 2b). And STING expression increased on D7, D14 compared with D0 and on P1 compared with P0 (*p* < 0.05) (Figure 2c).

In vitro, hDPSCs exposed to gradient LPS stimulation revealed higher viability at 10 and 20 μg/mL LPS stimulating for 1 and 3 days than that of the control group (*p* < 0.05) (Figure 2d), while a continuous overdrive of inflammatory stimulation led to cell death (Fawzy El‐Sayed et al. 2019). Simultaneously under 10 and 20 μg/mL LPS stimulation hDPSCs exhibited a significant reduction in ALP activity (*p* < 0.05) (Figure 2e,f) and less mineralized nodules formed (*p* < 0.05) (Figure 2g,h). Also, after hDPSCs treated by 20 μg/mL LPS for 1 day, the protein expression levels of the cGAS‐STING pathway including cGAS, STING and pTBK1 were markedly elevated (Figure 2i–l), along with a significant increase in the mRNA levels of downstream inflammatory cytokines IFN β, IL 6 and TNF α in the cGAS‐STING pathway (Figure S1b–d) (*p* < 0.05). Based on preliminary dose–response experiments assessing cell viability and odontogenic differentiation, we selected 20 μg/mL LPS for 1 day as the optimal condition to induce a moderate inflammatory response without compromising cell viability (Figure 2d–h). At this concentration, we observed significant activation of the cGAS‐STING pathway and upregulation of inflammatory cytokines, consistent with a physiologically relevant inflammatory microenvironment.

### 3.3 R‐Loops Magnified Inflammatory Level and Suppressed Odontogenic Differentiation of hDPSCs via cGAS‐STING Pathway

RT‐qPCR, Western blot and IF staining results confirmed overexpression of RNase H1 effectively downregulated R‐Loops in RNase H1 group compared with Empty Vector group (Figure 3a–e, Figure S1e,f) (*p* < 0.05). Simultaneously, the levels of molecules in cGAS‐STING axis (cGAS, STING, pTBK1, IFN β, IL 6, TNF α) decreased (Figure 3f–n) (*p* < 0.05). The OCN, RUNX2 and DMP1 mRNA upregulated in RNase H1 overexpression group after 20 μg/mL LPS treatment (*p* < 0.05) (Figure 3o–q). And ALP activity and mineralized nodules also increased compared with the Empty Vector group (Figure 3s–v). These results indicated that depletion of R‐Loops could alleviate inflammatory cytokines through downregulating cGAS‐STING signaling, which provided a more favorable condition for odontogenic differentiation of hDPSCs under inflammation.

**FIGURE 3:**
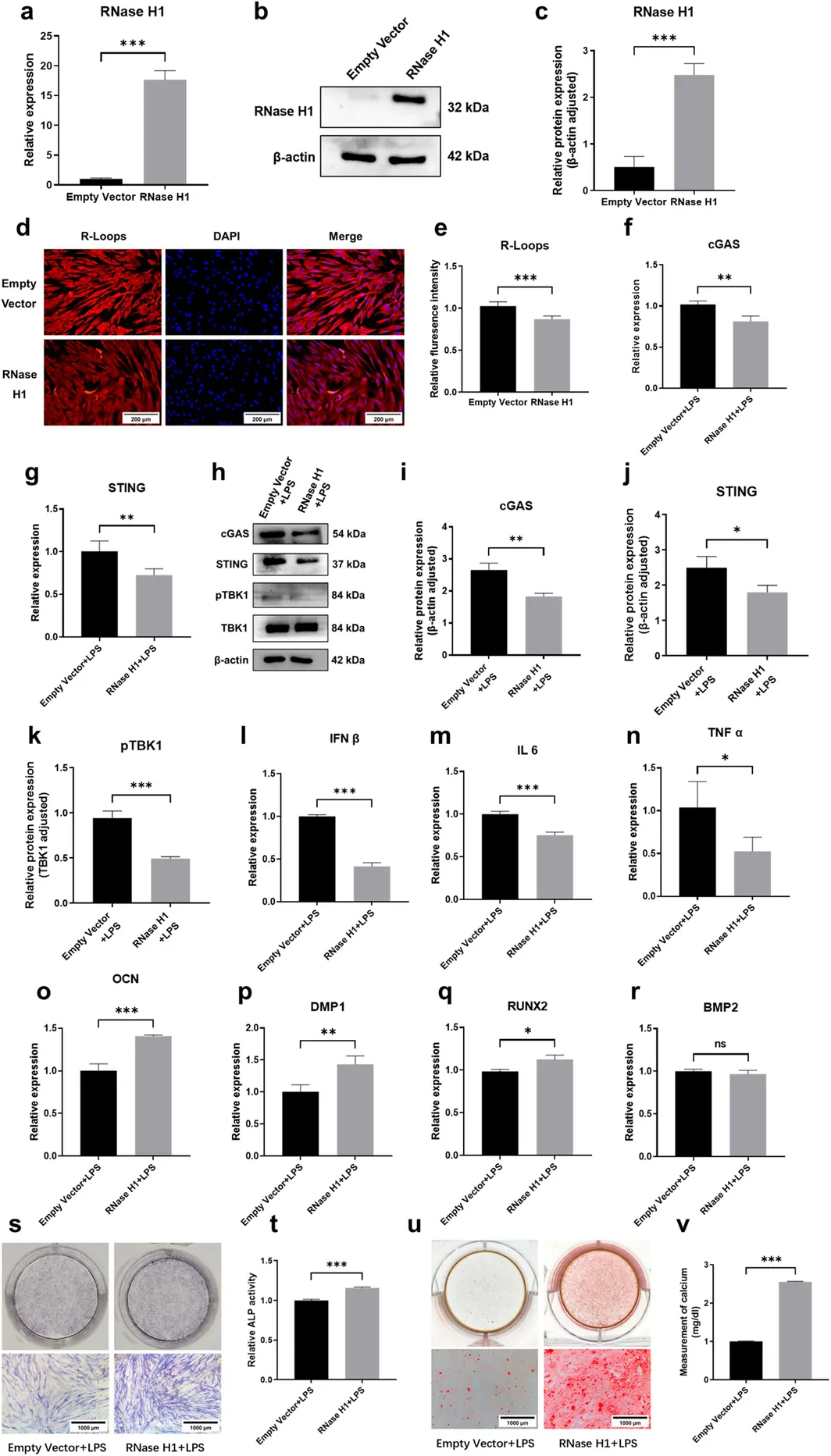
R‐Loops magnified inflammatory level and suppressed odontogenic differentiation of hDPSCs via cGAS‐STING pathway (a–c) The overexpression efficiency of RNase H1 in hDPSCs detected by RT‐qPCR and Western Blot. (d, e) IF staining of R‐Loops and quantitative analysis in Empty Vector group and RNase H1 group after hDPSCs treated by 20 μg/mL LPS for 1 day. (f–k) The gene and protein expression levels of cGAS‐STING pathway molecules (GAS, STING, pTBK1, TBK1) measured by RT‐qPCR and Western Blot in Empty Vector group and RNase H1 group after hDPSCs treated by 20 μg/mL LPS for 1 day. (l–n) The gene expression level of inflammatory cytokines (IFN β, IL 6, TNF α) measured by RT‐qPCR in Empty Vector group and RNase H1 group after hDPSCs treated by 20 μg/mL LPS for 1 day. (o–r) The gene expression level of odontoblastic differentiation‐related genes (OCN, DMP1, RUNX2, BMP2) in Empty Vector group and RNase H1 group measured by RT‐qPCR in Empty Vector group and RNase H1 group after hDPSCs treated by 20 μg/mL LPS for 7 days. (s, t) ALP staining and quantitative analysis of hDPSCs treated by 20 μg/mL LPS for 7 days in Empty Vector group and RNase H1 group. (u, v) Alizarin red S staining and quantitative analysis of hDPSCs treated by 20 μg/mL LPS for 21 days in Empty Vector group and RNase H1 group (significance was determined via *t*′‐test analysis of variance; data are represented as mean ± SD. ns *p* > 0.05, *p < 0.05, **p < 0.01, ***p < 0.001).

### 3.4 Suppressed cGAS‐STING Pathway Declined Inflammatory Cytokines and Rescued Odontogenic Differentiation of hDPSCs

H151, a selective and covalent STING antagonist, was induced to interrupt the cGAS‐STING pathway and 3 μM H‐151 was determined as the minimum effective concentration by measuring the downstream IFN β cytokine levels (Pan et al. 2021; Haag et al. 2018) (Figure 4a–c). The results showed H‐151 significantly attenuated the inflammatory cytokine levels (IFN β, IL 6, TNF α) (*p* < 0.05) (Figure 4d–f) but enhanced the odontogenesis‐related cytokines including OCN, DMP1 and BMP2 (*p* < 0.05) (Figure 4g–j). Also, blocking STING with H151 significantly promoted the ALP activity of hDPSCs and mineralized nodules formation compared with the control group (*p* < 0.05) (Figure 4k–n).

**FIGURE 4:**
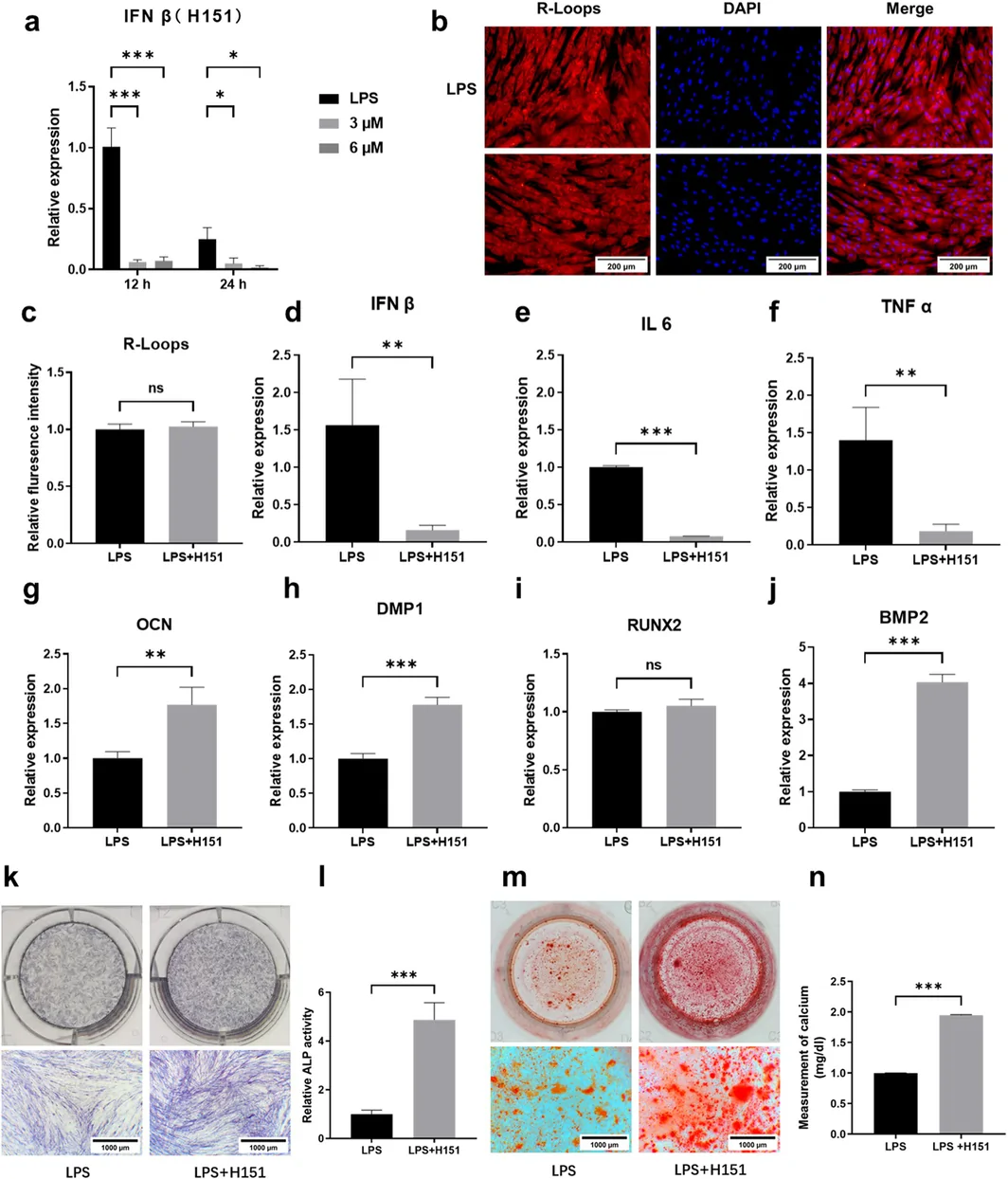
Suppressed cGAS‐STING pathway declined inflammatory cytokines and partly rescued odontogenic differentiation of hDPSCs (a) The mRNA expression level IFN β after hDPSCs treatment with 3 and 6 μM H151 for 12 and 24 h. (b, c) IF staining of R‐Loops and quantitative analysis in LPS and LPS + H151 group. (d–f) The gene expression level of inflammatory cytokines (IFN β, IL 6, TNF α) measured by RT‐qPCR after hDPSCs treated by 20 μg/mL LPS for 1 day with or without H151. (g–j) The gene expression levels of odontoblastic differentiation‐related genes (OCN, DMP1, RUNX2, BMP2) after hDPSCs treated by 20 μg/mL LPS for 7 days with or without H151. (k, l) ALP staining and quantitative analysis of hDPSCs treated by 20 μg/mL LPS for 7 days with or without H151. (m, n) Alizarin red S staining and quantitative analysis of hDPSCs treated by 20 μg/mL LPS for 21 days with or without H151 (significance was determined via one‐way ANOVA analysis and *t′*‐test analysis of variance; data are represented as mean ± SD. ns: *p* > 0.05, *p < 0.05, **p < 0.01, ***p < 0.001).

## 4 Discussion

The stability and genomic integrity of DNA are fundamental to cellular function, yet DNA is perpetually vulnerable to damage from external insults such as physical radiation and chemotherapy drugs, as well as endogenous threats like reactive oxygen species (ROS), replication errors, and base deamination (Ciccia and Elledge 2010). Among the most genotoxic forms of DNA damage are single‐ and double‐strand breaks (SSBs and DSBs), which are potent drivers of R‐Loops formation (Aguilera and Gómez‐González 2017). Dysregulation of R‐Loops formation and resolution has been implicated in various diseases, where aberrant immune signaling and genomic instability can arise from improper handling of these three‐stranded structures and then be detected by cGAS. Our experimental models of PDC injury revealed temporally correlated upregulation of γ H2AX (a marker for DNA damage and DSBs) and R‐Loops accumulation. The same findings were confirmed under LPS‐induced inflammation in vitro and this parallelism between in vivo and in vitro systems underscores the conserved nature of DNA damage–R‐Loops interactions.

The intricate interplay between unresolved mitotic DNA damage and inflammatory cascades warrants particular attention. Typically, inflammation triggers the production of reactive oxygen and nitrogen species (RONS), leading to mitochondrial dysfunction and DNA oxidation, thereby activating the DNA damage response (DDR) pathway while simultaneously impairing DNA repair mechanisms (Pezone et al. 2023). This creates a pernicious cycle where persistent DDR signaling amplifies inflammatory cytokine production through the cGAS‐STING and NF‐κB pathway (Zhao et al. 2023; Higo et al. 2017). The critical function of R‐Loops was unveiled as a key regulator of inflammatory homeostasis, serving as a gatekeeper of the cGAS‐STING inflammatory cascade. Our findings position R‐Loops as critical molecular rheostats in PDC injury and regeneration, demonstrating that lentivirus‐mediated RNase H1 expression effectively attenuates cGAS‐STING signaling and this intervention not only reduced inflammatory markers but also restored the odontogenic differentiation capacity of hDPSCs. These results indicate that understanding the role of R‐Loops in the cGAS‐STING pathway is important for elucidating the molecular basis of immune and inflammatory responses in PDC injury and regeneration and R‐Loops regulation may be a potential therapeutic node for breaking the pathogenic feedback loop.

cGAS‐STING axis is characterized as an evolutionarily highly conserved mechanism of immunity, which can be activated in dental pulpitis and magnifies inflammation via activating IFN‐I and NF‐κB pathway (Tian et al. 2022). Besides residing in the cytosol and surveying for cytosolic microbial DNA, cGAS can also be enriched on centromeric satellite DNA in the nucleus depending on the non‐enzymatic N‐terminal domain, suggesting it may tether to nuclear R‐Loops (Gentili et al. 2019; Jiang et al. 2019). Recently, researchers have reported that excessive R‐Loops trigger innate immune response and inflammatory response via the cGAS‐STING pathway in different diseases, including Aicardi‐Goutières syndrome (Cristini et al. 2022), chronic pancreatitis (Chatzidoukaki et al. 2021), and amyotrophic lateral sclerosis (ALS) (Islam et al. 2024). However, the relationship between R‐Loops and cGAS‐STING in dental contexts remains unexplored. Our experiments revealed elevated cGAS and STING levels in PDC damage models and LPS‐treated hDPSCs. Overexpression of RNase H1, which resolves R‐Loops, significantly downregulated the cGAS‐STING axis and inflammatory cytokines, further providing mechanistic evidence for R‐Loops‐mediated immune activation in pulpitis.

An important question arising from our study is how nuclear R‐Loops engage cytoplasmic cGAS. Recent mechanistic studies provide compelling insights. Crossley et al. (2023) demonstrated that upon R‐Loops dysregulation, XPG/XPF‐dependent processing generates excised RNA–DNA hybrids that are actively exported to the cytoplasm via CRM1‐mediated nuclear transport, where they activate cGAS and TLR3. Similarly, Chatzidoukaki et al. (2021) showed in DNA repair‐deficient models that R‐Loops trigger RPA‐dependent release of ssDNA fragments into the cytoplasm. These findings establish that R‐Loops or their processed products can exit the nucleus through active mechanisms, not merely passive leakage. In our inflammatory model, RNase H1 overexpression reduced both R‐Loops and cGAS‐STING activation, while STING inhibition did not affect R‐Loops levels, positioning R‐Loops upstream of this pathway. Although direct visualization of R‐Loops translocation in hDPSCs warrants future investigation, the existing evidence supports a model where inflammation‐induced R‐Loops undergo nucleolytic processing and active export to trigger cytosolic cGAS‐STING signaling.

Emerging evidence suggests that R‐Loops are involved in stem cell differentiation, with dynamic R‐Loops turnover linked to the activation of pluripotency and lineage‐specific genes (Yan et al. 2020). Besides, the dynamic turnover of R‐Loops subset was accompanied by the activation of the pluripotent transcriptional regulatory network (TRN) which was confirmed to be positively correlated with gene expression during reprogramming (Li et al. 2022). Similarly, STING has been implicated in bone metabolism. Some researchers found that up‐regulation of STING expression under heavy mechanical force conditions significantly increased osteoclast activity and accelerated orthodontic tooth movement (Zhu et al. 2025). Further, upregulation of STING expression caused by aging led to HK2 separation from VDAC1, leading to the opening of the mitochondrial inner mitochondrial permeability transition pore (mPTP), mitochondrial dysfunction and abnormal osteogenic differentiation, thereby disrupting bone homeostasis (Cai et al. 2024). These converging lines of evidence imply that both R‐Loops and STING may orchestrate odontogenic differentiation, though their precise interplay within inflammatory niches requires further investigation. Particularly intriguing is the potential crosstalk between DNA topology‐mediated epigenetic regulation and metabolic reprogramming in stem cell fate decisions.

Inflammatory response constitutes a dual‐edged sword in the PDC regenerative processes. Moderate stimulus facilitates the removal of necrotic tissue and promotes hDPSCs differentiation through cytokines like TNF α and ROS (Kim et al. 2023; Cooper et al. 2011, 2014). But intense insults affect the cells' fate as well as the outcome of cell‐mediated tissue reparative or regenerative endeavors (Fawzy El‐Sayed et al. 2019; Yang et al. 2021; Cooper et al. 2017). Clinically persistent noxious stimulus including bacteria, mechanical stimulus, and dental operation may induce dental pulp inflammation, PDC injury, and irreversible pulpitis (Sequeira et al. 2023). Therefore, it is urgent and meaningful to control inflammation at a level that facilitates odontogenic differentiation of hDPSCs and promotes PDC regeneration under an inevitable inflammatory microenvironment.

Our results hinted that intervening R‐Loops or cGAS‐STING axis may offer a viable strategy to modulate inflammation and create a favorable microenvironment for odontogenic differentiation. It aligns with previous reports that cGAS‐STING pathway inhibition reversed the innate immune response and inflammatory responses (Zhao et al. 2024; Pan et al. 2024). Moreover, mis‐memory R‐Loops generated during cell reprogramming were probably linked to metabolic abnormality, which further reinforced the connection between epigenetic memory and metabolic memory (Yan et al. 2020). Therefore, harnessing unscheduled R‐Loops may be a feasible method for improving reprogramming quality and generating more suitable iPSCs for regenerative endeavor. However, there are still some doubts that need to be resolved. During the period of odontogenic differentiation of hDPSCs, does the R‐Loops level fluctuate or if they participate in the odontogenesis‐related genes regulation? Eventually how to resolve pathological R‐Loops to maintain gene integrity but avoid damaging physiological one? Critical questions remain regarding R‐Loops spatiotemporal dynamics during odontogenic differentiation and the precise demarcation between physiological versus pathological R‐Loops populations—challenges requiring advanced single‐cell sequencing and spatial transcriptomics for resolution.

The identification of the R‐Loops/cGAS‐STING axis as a critical regulator linking inflammation to impaired odontogenic differentiation offers a potential therapeutic target for regenerative endodontics (Figure 5). Pharmacological modulation of this pathway using RNase H1 or STING inhibitors may attenuate excessive inflammation while preserving hDPSCs differentiation capacity. Localized delivery of such agents could represent a promising adjunctive strategy to promote pulpal repair following deep caries or traumatic injury. Nevertheless, we wish to acknowledge several limitations in our study, along with our plans to address them in future work. First, while we used RNase H1 overexpression and STING inhibition to establish a causal relationship, direct genetic knockdown of cGAS or STING would provide even stronger mechanistic evidence, which we intend to pursue in subsequent investigations. Second, our in vivo findings are derived from a single animal species with a limited number of time points; validation in human pulpitis specimens is therefore an essential next step, and we have already outlined such experiments in our revised Discussion. Third, the precise mechanism by which R‐loops translocate to the cytoplasm to activate cGAS was not directly visualized in this study. We recognize this as an important avenue for future research and are currently exploring approaches—such as high‐resolution imaging and subcellular fractionation—to directly capture this translocation process. Fourth, while we assessed odontogenic differentiation by ALP activity, Alizarin Red S staining, and qPCR of differentiation‐related genes (OCN, DMP1, RUNX2, BMP2), protein‐level validation of key markers such as DMP1 or DSPP was not performed. This constitutes a limitation of the present study, and future investigations will include Western blot or immunofluorescence to confirm these findings at the translational level.

**FIGURE 5:**
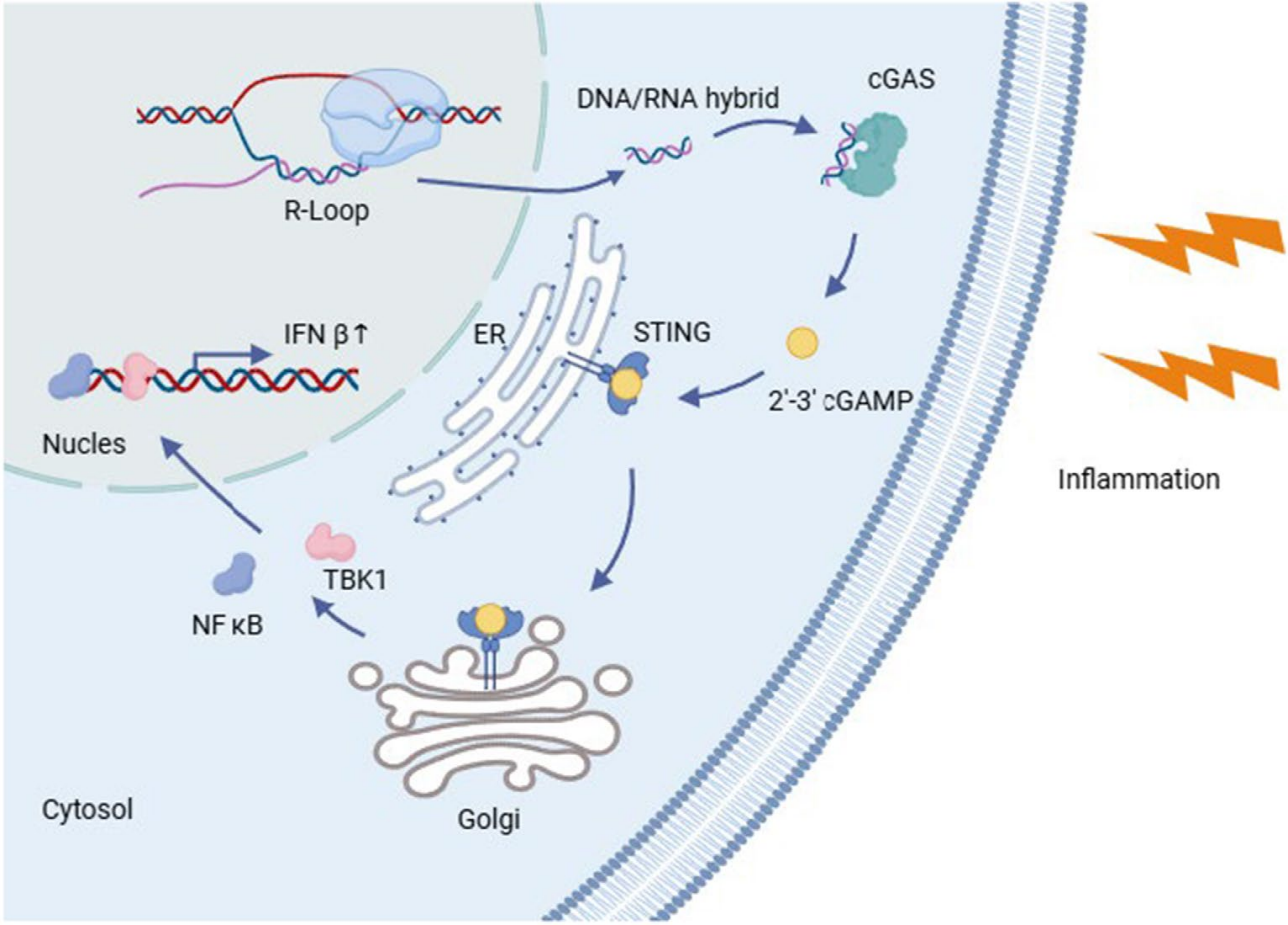
Excessive R‐Loops induced by inflammation activated cGAS‐STING axis (image was created with BioRender.com).

## 5 Conclusion

This study establishes R‐Loops/cGAS‐STING axis dysregulation as a pivotal mechanism linking inflammation and impaired odontogenesis in PDC injury. Mechanistically, inflammatory stimuli induce pathological R‐Loops accumulation that activates cGAS‐STING signaling, creating a self‐perpetuating inflammatory cycle detrimental to hDPSCs differentiation. Therapeutic interventions targeting either R‐Loops (via RNase H1) or cGAS‐STING signaling effectively attenuated inflammation and partially restored odontogenic potential, highlighting this axis as a promising therapeutic target to promote PDC complex regeneration.

## Author Contributions

**Yiqing Wang:** conceptualization, project administration, software, methodology, writing – original draft. **Zhipu Luo:** investigation, project administration, data curation. **Yun Yang:** conceptualization, methodology, software, data curation, validation, writing – original draft, formal analysis, project administration. **Yating Miao:** data curation, project administration, investigation. **Lin Niu:** visualization, resources, writing – review and editing. **Ruirui Liu:** writing – review and editing, funding acquisition, resources, supervision, validation.

## Funding

This work was supported by the key project of the Natural Science Foundation of Shaanxi Province (2022JZ‐56).

## Disclosure

AI declaration: We did not use AI‐generated work in this manuscript.

## Ethics Statement

The research project you applied “R‐Loops mediated odontogenic differentiation of hDPSCs by activating the cGAS‐STING pathway under inflammatory microenvironment” was reviewed by the Medical Ethics Committee of Stomatology Hospital of Xi'an Jiaotong University. The project accords with the requirements of medical and teaching ethics and is approved to conduct. The ethics approval reference number for the study is [2022]005, and the date of the ethics approval was May 10, 2022.

## Consent

This research obtained the consent of all patients.

## Conflicts of Interest

The authors declare no conflicts of interest.

## Supporting information


**Figure S1:** (a) GO enrichment analysis of the differentially expressed genes between control and LPS group (20 μg/mL for 1 day). (b–d) The gene expression level of inflammatory cytokines (IFN β, IL 6, TNF α) stimulated by different concentration of LPS (0, 10, 20 μg/mL) for 1, 3, and 5 days. (e, f) IF staining of R‐Loops and quantitative analysis in Empty Vector group and RNase H1 group.

## Data Availability

The data that support the findings of this study are available on seasonable request by contacting the corresponding author. All additional files are included in the manuscript.
