# Supplementary material for: R‐Loops Mediated Odontogenic Differentiation of hDPSCs by Activating the cGAS‐STING Pathway Under Inflammatory Microenvironment
Source: Oral Dis. 2026 Jun 12;32(10):3291–302. doi: 10.1111/odi.70379 (PMC13768930; doi:10.1111/odi.70379)
Supplement: Supplementary file 1 [file ODI-32-3291-s001.docx]

**Supplement:**

**a**

**b-d**

**e-f**

(a)GO enrichment analysis of the differentially expressed genes between control and LPS group (20 μg/mL for 1 d).

(b-d) The gene expression level of inflammatory cytokines (IFN β, IL 6, TNF α) stimulated by different concentration of LPS (0, 10, 20 μg/mL) for 1 d, 3 d and 5 d.

(e-f) IF staining of R-Loops and quantitative analysis in Empty Vector group and RNase H1 group.
